# SaaS sRNA promotes *Salmonella* intestinal invasion via modulating MAPK inflammatory pathway

**DOI:** 10.1080/19490976.2023.2211184

**Published:** 2023-05-09

**Authors:** Linlin Cai, Yunting Xie, Liangting Shao, Haijing Hu, Xinglian Xu, Huhu Wang, Guanghong Zhou

**Affiliations:** Jiangsu Collaborative Innovation Center of Meat Production and Processing, Quality and Safety Control, Nanjing Agricultural University, Nanjing, P.R. China

**Keywords:** *Salmonella* Enteritidis, sRNA, virulence, intestinal barrier, gut microbiota, MAPK pathway

## Abstract

*Salmonella* Enteritidis is a foodborne enteric pathogen that infects humans and animals, utilizing complex survival strategies. Bacterial small RNA (sRNA) plays an important role in these strategies. However, the virulence regulatory network of *S*. Enteritidis remains largely incomplete and knowledge of gut virulence mechanisms of sRNAs is limited. Here, we characterized the function of a previously identified *S**almonella* adhesive-associated sRNA (SaaS) in the intestinal pathogenesis of *S*. Enteritidis. We found that SaaS promoted bacterial colonization in both cecum and colon of a BALB/c mouse model; it was preferentially expressed in colon. Moreover, our results showed that SaaS enhanced damage to mucosal barrier by affecting expressions of antimicrobial products, decreasing the number of goblet cells, suppressing mucin gene expression, and eventually reducing thickness of mucus layer; it further breached below physical barrier by strengthening invasion into epithelial cells in Caco-2 cell model as well as decreasing tight junction expressions. High throughput 16S rRNA gene sequencing revealed that SaaS also altered gut homeostasis by depleting beneficial gut microbiota while increasing harmful ones. Furthermore, by employing ELISA and western blot analysis, we demonstrated that SaaS regulated intestinal inflammation through sequential activation P38-JNK-ERK MAPK signaling pathway, which enabled immune escape at primary infection stage but strengthened pathogenesis at later stage, respectively. These findings suggest that SaaS plays an essential role in the virulence of *S*. Enteritidis and reveals its biological role in intestinal pathogenesis.

## GRAPHICAL ABSTRACT

**Figure:**
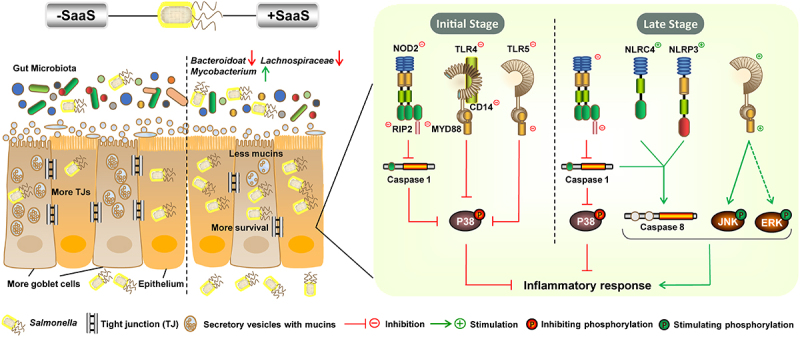

## Introduction

As a food-borne pathogen, *Salmonella* is responsible for a wide range of enteric diseases from mild, self-limiting gastroenteritis to life-threatening systemic infection. The symptoms of the gastroenteritis caused by *Salmonella enterica* Serovar Enteritidis (*S*. Enteritidis) are non-bloody vomiting and nausea, accompanied by abdominal pain, myalgia, headache and arthralgia in people of all ages.^1,2^ What’s more, for those immunocompromised such as children, older and patients, severe diseases like bacteremia and meningitis can be observed.^3^ This wide range of clinical pictures makes *Salmonella* a continuous threat that leads to high disease burden around the world especially in Africa followed by Southeast Asia.^4,5^ *S*. Enteritidis, a facultative intracellular Gram-negative pathogen, is one of the most common serotypes of *Salmonella*. After ingestion it hyper-replicates within its host’s gut tissue causing inflammation, which then reaches mesenteric lymph nodes or further systemic sites such as liver and spleen.^6,7^ Breaching the intestinal barrier is thought to be the initial step of the *S*. Enteritidis infection.^8^ Since animal intestine contains natural microbiota which suppress pathogen growths by competing for nutrients, mucosal barriers preventing pathogens from invading epithelium and physical barriers separating pathogens from internal milieu,^9^ any damage done to these barriers will lead to increased bacterial dissemination reinstating its virulence.^10^ Thus, understanding how pathogens break through these barriers is essential for avoiding microbial infections or their progression.

To breach the intestinal barrier, *Salmonella* employs a variety of virulence factors, among which *Salmonella* pathogenicity island (SPI)-1 and SPI-2 effectors are predominantly studied. SPI-1 effectors like SopA, SopB and SopE act on actin dynamics, chemokine secretion, and tight junction (TJ) formation during early phase of infection,^11^ whereas SPI-2 effectors such as SseF and SseG take over post-intracellular growth and survival.^12^ Other factors include CsgB and PegD important for colonization^13^ and AvrA inhibiting JNK MAPK pathway, thus inhibiting inflammatory response and stabilizing tight junctions.^14,15^ Despite the fact that many of these factors have been well investigated, the regulatory network of *S*. Enteritidis is still substantially incomplete, and new regulators are constantly being discovered.

Small non-coding RNAs (sRNAs) have been identified in recent years as post-transcriptional regulators that can have a vital effect on diverse cellular processes, such as sRNA4130247 for carbon starvation,^16^ CyaR for hyperosmotic stress and DsrA for acid stress.^17^ *In vivo* conditions are complex and diverse, so sRNAs are thought to play an important role in *Salmonella* survival and pathogenesis. To date, only a few sRNAs have been found to regulate virulence out of hundreds of candidates in *Salmonella*; most belong to *S*. Typhimurium. Research has mostly focused on cell models such as macrophages or epidermic cells to understand possible roles in virulence. To name a few, IsrM, RyhBs, and STnc640 were found involved in bacterial invasion of epithelial cells or intracellular replication and survival in macrophages.^18–20^ Further *in vivo* studies are limited, with most stopping with the lethality of animals and bacterial colonization.^18^ Overall, there is still much more research needed to better understand the actual functions of sRNAs in *Salmonella* pathogenesis *in vivo*, particularly within the intestine.

Previous study revealed that *S**almonella* adhesive-associated sRNA (SaaS) promotes *Salmonella* virulence by enhancing mortality, systemic inflammation and bacterial dissemination in the intestine.^21^ However, the role of SaaS in *S*. Enteritidis intestinal pathogenicity remains unclear. This study aims to investigate how SaaS contributes to the complex interactions among luminal microbes, physical barrier and mucosal barrier for better understanding of its biological role in *S*. Enteritidis intestinal pathogenicity. Such knowledge is expected to offer insights into development of therapies for bacterial infections and help refine the *Salmonella* virulence regulatory network.

## Results

### SaaS is a colon-responsive sRNA that promotes *Salmonella* colonization of the mouse intestine

**Figure 1.**
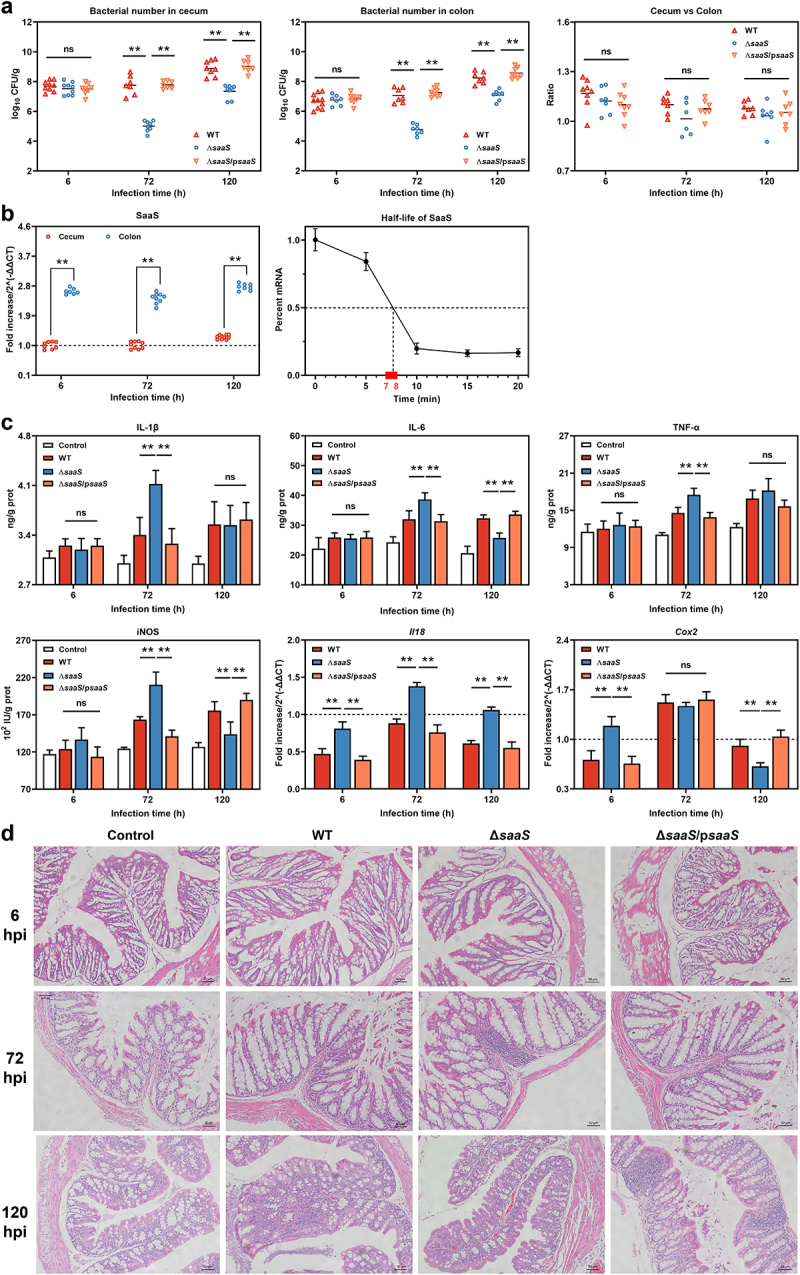
Bacterial dissemination and inflammatory response induced by SaaS *in* *vivo*. (a) Bacterial colonization of WT, Δ*saaS* and Δ*saaS*/p*saaS* in cecum and colon, and the ratio between bacterial colonization in cecum and bacterial colonization in colon in one mouse; (b) Expression of SaaS in colonized *Salmonella* in cecum and colon, and half-life of SaaS in the simulated intestinal environment; (c) The concentration of IL-1β, IL-6, TNF-α and iNOS and the expressions of *Il18* and *Cox2* mRNAs in colon; (d) Representative H&E-stained colonic tissue sections of WT, Δ*saaS* and Δ*saaS*/p*saaS*-infected mice (Scale bars, 50 μm). For mRNA expression, the control was set to 1 and indicated by dashed line. Data are represented as means±SD; n = 7–10. Statistical significance was assessed using Student’s *t*-test. *P < 0.05, **P < 0.01.

To examine the effects of SaaS on *Salmonella* adherence, we carried out *in vivo* mouse colonization experiments. The wild strain WT-infected mice and complement strain Δ*saaS*/p*saaS*-infected mice presented similar changes in this assay. As shown in Figure 1a, while the number of CFUs recovered from colon and cecum was significantly higher (*P* < 0.01) in the WT group than in the Δ*saaS* group at 72 and 120 hpi, a dramatic decrease was observed for *Salmonella* numbers within the latter at 72 hpi compared with 6 hpi. Interestingly, even though the sustaining accumulation makes cecum harbors the most microbiota in the gastrointestinal tract and more *Salmonella* colonization here, there was no significant difference between WT-infected mice and Δ*saaS*-infected mice in terms of the ratio of bacteria colonizing the cecum versus the colon, indicating a possible rule of *Salmonella* colonization distribution throughout the intestine. For SaaS itself, the results showed that the half-life of SaaS was about 7–8 min, and levels of SaaS were 2.2–2.6-fold greater within colonic tissue than cecal samples (Figure 1b). These results indicate that SaaS, activated by colonic environment, is a positive regulator of *Salmonella* colonization.

### SaaS promotes the time-dependent regulation of intestinal inflammatory response

The present study investigated whether SaaS mediated the secretion of cytokines *in vivo*, with the interleukin (IL)-1β, IL-6, IL-18, tumor necrosis factor-α (TNF-α), inducible nitric oxide synthase (iNOS) and cyclooxygenase-2 (COX-2) levels in colon after infection examined with ELISA or RT-qPCR (Figure 1c). At 6 hpi, there was no significant difference found between all groups for the levels of IL-1β, IL-6, TNF-α and iNOS, while only *Il18* and *Cox2* mRNA were higher in the Δ*saaS* group than that of WT group. At 72 hpi all cytokine levels except *Cox2* were significantly higher in the Δ*saaS* group compared to those observed from the WT group. Conversely at 120 hpi, except for increased *Il18* mRNA expression, lower levels of IL-6, iNOS and *Cox2* were detected in the Δ*saaS* group than in the WT group, indicating a time-dependent response. In addition, H&E staining showed that although no serious pathological syndromes were observed, the histopathological appearance in the colon exhibited significant differences among groups (Figure 1d). Compared with the WT group, the Δ*saaS* group displayed more inflammatory infiltration at 72 hpi. At 120 hpi, an overall enhancement of inflammatory response was evident; however, compared to that in the Δ*saaS* group, those infected with WT strain had a more intense infiltration of inflammatory cells. This tendency was consistent with the trends of tested cytokines (Figure 1c). These results indicated that intestinal inflammation is mediated by SaaS in a time-dependent manner.

### SaaS contributes to intestinal mechanical and mucosal barrier dysfunction

**Figure 2.**
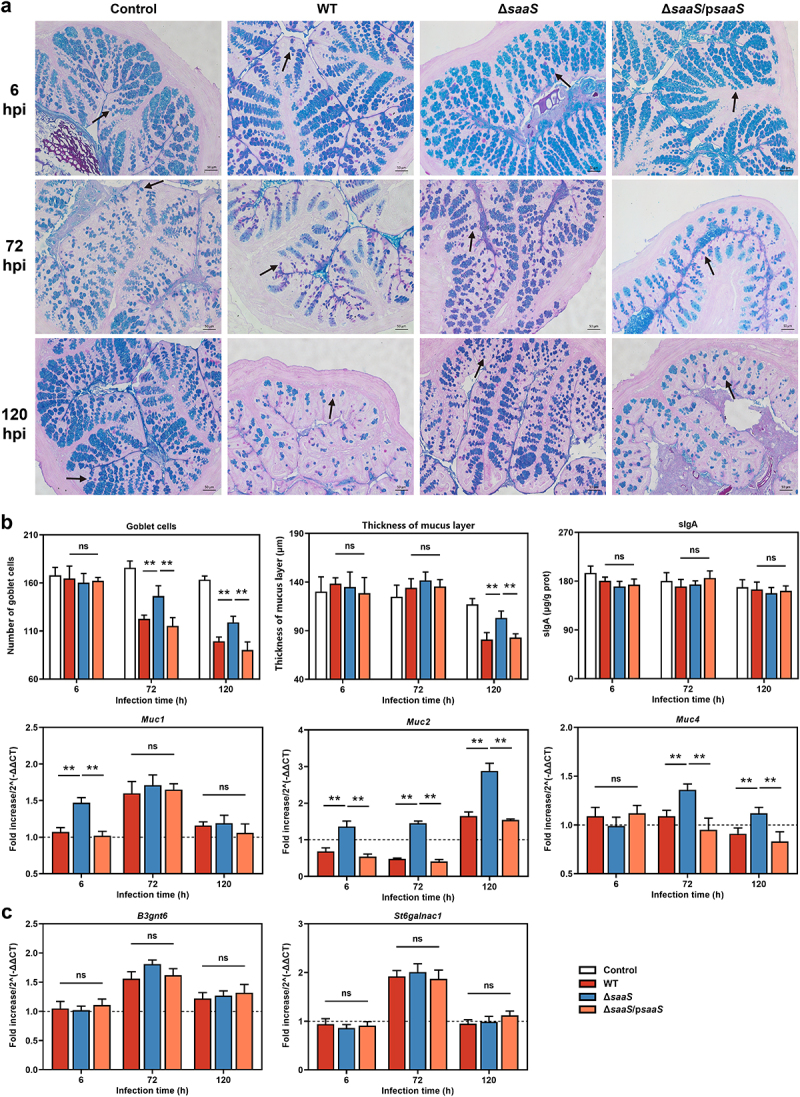
Effects of SaaS on the colonic mucus barrier. (a) Representative AB/PAS-stained colonic tissue sections of WT, Δ*saaS* and Δ*saaS*/p*saaS*-infected mice (Scale bars, 50 μm); (b) The number of goblet cells (marked with a black arrow), the thickness of mucus layer, sIgA levels and the expressions of *Muc1/2/4* mRNA; (c) Expressions of mucin biosynthesis genes including *B3gnt6* and *St6galnac1*. For mRNA expression, the control was set to 1 and indicated by dashed line. Data are represented as means±SD; n = 7–10. Statistical significance was assessed using Student’s *t*-test. *P < 0.05, **P < 0.01.

The integrity of the intestinal barrier is essential for maintaining intestinal immune homeostasis and activating a proper immune response to pathogenic bacteria, with mucosal and physical barriers playing important roles.^22^ Therefore, we further analyzed the effect of *Salmonella* mediated by SaaS on the colonic mucosal and physical barrier *in vivo*. The relative number of goblet cells, mRNA expressions of mucin, thickness of mucus layer and secretory immunoglobulin A (sIgA) levels were all combined to thoroughly evaluate the effects of SaaS on the mucosal barrier (Figure 2). As shown in Figure 2a-b, at 72 and 120 hpi, respectively, the relative number of goblet cells was significantly higher (*P* < 0.01) in the Δ*saaS* group than that in the WT group. The virulence factor SopB is the only reported one responsible for regulating the death of goblet cells, which *Salmonella* uses to inhibit the process of necroptosis in goblet cells.^23^ Hence we want to know whether SaaS utilizes SopB to lead to the death of goblet cells. Nevertheless, results showed that the level of *sopB* mRNA in the WT strain was significantly higher than that in the Δ*saaS* group (Fig S1), which implies a different mechanism. Meanwhile, gene expression analysis revealed that in the Δ*saaS* group, *Muc1* (encoding Mucin-1) had higher expression at 6 hpi; *Muc2* (encoding Mucin-2) had higher expression throughout infection; while *Muc4* (encoding Mucin-4) had higher expression at 72 hpi and 120 hpi (Figure 2b). Furthermore, the expressions of mucin biosynthesis genes *B3gnt6* (encoding core 3 1,3-N-acetylglucosaminyltransferase) and *St6galnac1* (encoding alpha-N-acetylgalactosaminide alpha-2,6-sialyltransferase 1) were evaluated. There was no significant difference among the groups (Figure 2c), implying that *Salmonella* has no effect on mucin biosynthesis through SaaS. Hence, it’s the death of goblet cells rather than a decrease in mucin biosynthesis that is responsible for the decreased mucin. As expected, the thickness of mucus layer in the Δ*saaS* group was significantly higher than that in the WT group at 120 hpi, indicating an inhibitory role played by SaaS on host’s recovery from damaged mucosal barrier.

**Figure 3.**
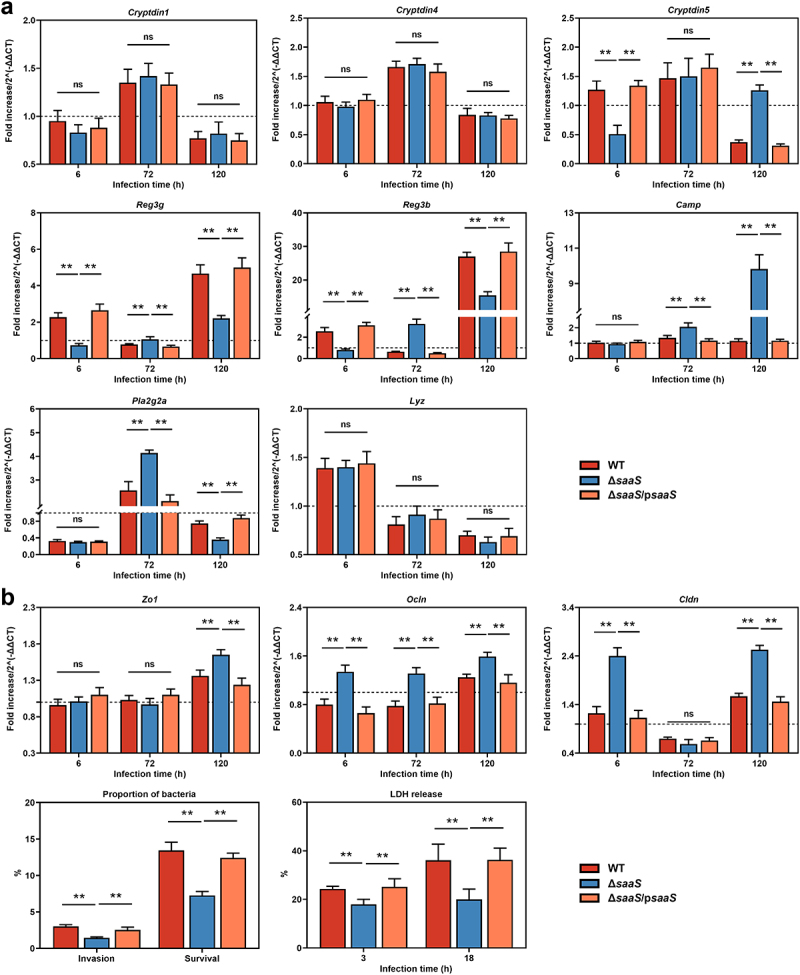
Effects of SaaS on the colonic antimicrobial products and physical barrier. (a) Expressions of antimicrobial product genes including *Cryptdin1/4/5*, *Reg3g*, *Reg3b*, *Pla2g2a*, *Camp*, and *Lyz*; (b) Invasion and survival of *S*. Enteritidis to Caco-2 cells, LDH release of Caco-2 cells treated with *S*. Enteritidis and the expressions of *Zo1*, *Ocln* and *Cldn* mRNA. For mRNA expression, the control was set to 1 and indicated by dashed line. Data are represented as means±SD; n = 7–10. Statistical significance was assessed using Student’s *t*-test. *P < 0.05, **P < 0.01.

Antimicrobial products (AMPs), including alpha defensins (Cryptdins encoded by *Cryptdin1/4/5*), C-type lectin regenerating islet-derived protein 3 (RegIIIγ encoded by *Reg3g* and RegIIIβ encoded by *Reg3b*), secretory phospholipase A2 type IIA (sPLA2 encoded by *Pla2g2a*), cathelicidins (encoded by *Camp*), and lysozymes (encoded by *Lyz*) are released by Paneth cells or goblet cells to provide critical local bactericidal activity protecting the intestinal mucosa.^24^ Throughout the invasion, there was no significant difference between groups in *Cryptdin1/4* and *Lyz* in this study (Figure 3a). *Cryptdin5*, *Reg3g*, and *Reg3b* mRNA levels were significantly lower (*P* < 0.01) in the Δ*saaS* group compared to the WT group at 6 hpi; however, antimicrobial product expressions showed an opposite trend at 72 hpi, with *Reg3g*, *Reg3b*, *Camp* and *Pla2g2a* mRNAs being higher in the Δ*saaS* group. This trend is maintained, with *Cryptdin5* and *Camp* mRNAs significantly higher (*P* < 0.01) in the Δ*saaS* group, but *Reg3g*, *Reg3b* and *Pla2g2a* mRNAs decreasing.

The physical barrier, which is made up of intestinal epithelium and tight junctions (TJs), serves as a secondary protective layer following the mucus layer.^25^ Dysfunction of TJs or intestinal epithelium often results in increased intestinal permeability, known as leaky gut, which promotes the risk of microbial infection and tissue injury.^26,27^ In mice infected with Δ*saaS* strain, compared to those infected with WT strain, significantly higher gene expression levels of occludin (*Ocln*) were observed at all times, claudin (*Cldn*) except at 72 hpi and zonula occludens-1 (*Zo1*) at 120 hpi from the point of TJs that known as the paracellular route (Figure 3b). To further investigate the effect of SaaS on intestinal permeability, Caco-2 monolayer was used as an *in vitro* model that mimics the transcellular route. As shown in Figure 3b, CFU recovered from Caco-2 cells was approximately 1.8 fold higher (*P* < 0.01) in the WT group than in the Δ*saaS* group for both invasion and intracellular survival ability. Correspondingly, lactate dehydrogenase (LDH) activities measured in all WT-infected Caco-2 cells were much greater (*P* < 0.05) than those infected with Δ*saaS*. These data suggest that SaaS contributes to increased intestinal permeability and translocation of *S*. Enteritidis across the intestinal barrier.

### SaaS contributes to intestinal biological barrier damage by altering gut microbiota ecology and function

Trillions of bacteria reside on the mucosal and epithelial surfaces of the host intestine, maintaining a relatively stable composition while principally coexisting with the host and interacting with other barriers.^28^ To access the microbial diversity associated with *Salmonella* strains in colonic microbiota, we estimated alpha- and beta-diversity.

#### Richness and diversity of microbiota

**Figure 4.**
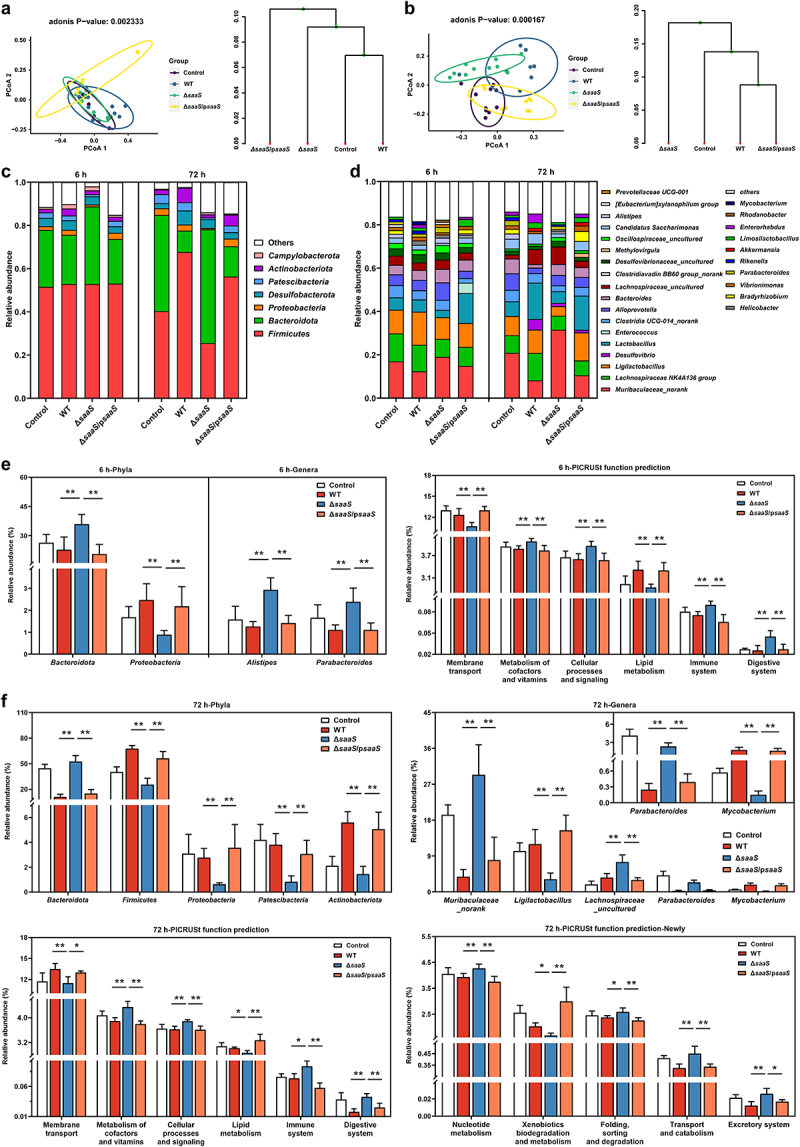
Effects of SaaS on the diversity of colon microbiota. (a) Principal coordinate analysis (PCoA) and clustering analysis at 6 hpi; (b) PCoA and clustering analysis at 72 hpi; (c) Composition of colon microbiota at the phyla levels at 6 and 72 hpi; (d) Composition of colon microbiota at the genera levels at 6 and 72 hpi. (e) Differential microbiota at phyla and genera levels and functional prediction of colonic microbial genes at 6 hpi; (f) Differential microbiota at phyla and genera levels and functional prediction of colonic microbial genes at 72 hpi. hpi: hours after infection. Data are represented as means±SD; n = 7–10. Statistical significance was assessed using Student’s *t*-test. *P < 0.05, **P < 0.01.

The Chao l and ACE indexes are used to positively measure the richness of microbial communities.^29,30^ The Shannon and Simpson indexes are employed to assess species diversity positively, and evenness negatively, respectively.^31^ Results showed that values of ACE and Chao1 in the Δ*saaS* group were significantly higher than those in other infection groups. The Shannon index for the Δ*saaS* group was also higher than in the WT group (Table 1). However, no difference was observed for the Simpson index in the colon at 6 hpi. To gain a better understanding of microbial composition, the principal coordinate analysis (PCoA) and clustering analysis were conducted.^32^ As shown in Figure 4a-b, gut microbial communities in mice infected with Δ*saaS* differed significantly from those in mice infected with WT, Δ*saaS*/p*saaS* or control. Interestingly, changes in gut microbial communities occurred also during infection with Δ*saaS*/p*saaS* at 6 hpi. Overall, these findings reveal that SaaS contributes to the alteration of gut microbial richness and diversity.

**Table 1.** Alpha diversity characteristics of gut microbiota.

Alpha diversity	0.97				
	Time (h)	Control	WT	Δ*saaS*	Δ*saaS*/p*saaS*
Chao1	6	1624.70 ± 65.61^b^	1246.40 ± 191.12^c^	1795.72 ± 88.11^a^	1224.49 ± 231.86^c^
	72	1373.35 ± 68.51^a^	896.01 ± 85.06^b^	1548.98 ± 211.34^a^	915.38 ± 171.15^b^
ACE	6	1673.02 ± 75.58^b^	1470.67 ± 136.65^c^	1832.61 ± 94.08^a^	1241.54 ± 249.91^c^
	72	1363.30 ± 76.04^a^	936.55 ± 68.87^c^	1174.29 ± 121.63^b^	983.58 ± 57.53^c^
Shannon	6	6.85 ± 0.21^a^	5.88 ± 0.49^b^	7.15 ± 0.26^a^	6.05 ± 0.73^b^
	72	6.55 ± 0.23^a^	6.16 ± 0.23^b^	6.91 ± 0.21^a^	5.70 ± 0.32^c^
Simpson	6	0.034 ± 0.003^a^	0.026 ± 0.009^b^	0.028 ± 0.006^b^	0.026 ± 0.004^b^
	72	0.032 ± 0.004^a^	0.037 ± 0.002^a^	0.025 ± 0.004^b^	0.037 ± 0.004^a^

Data represents as mean ± standard deviation; different lowercase letters in the same line indicate significant difference (*P* < 0.05). Statistical significance was assessed using one-way ANOVA.

#### Composition and functional prediction of microbiota

Significant changes induced by SaaS were observed at both phylum and genus levels. At the phylum level (Figure 4c), *Firmicutes* was found to be the most prevalent in all groups, followed by *Bacteroidota* and *Desulfobacteria* at 6 hpi, while at 72 hpi *Bacteroidota* overtook *Firmicutes* as the main microbiota in the Δ*saaS* group. At the genera level (Figure 4d), *Muribaculaceae_norank*, *Lachnospiraceae NK4A136 group*, *Ligilactobacillus* and *Desulfovibrio* were among the top four genera. The differential phyla and genera at both infection time points were next analyzed by comparing all groups (Figure 4e-f). At 6 hpi, the Δ*saaS* group had a significantly higher relative abundance of *Bacteroidota* and lower relative abundance of *Proteobacteria* compared to the WT group. *Alistipes* and *Parabacteroides* belonging to *Bacteroidota* were more abundant in the Δ*saaS* group than in the WT group (Figure 4e). At 72 hpi, similar results including higher abundance of *Bacteroidota* and lower *Proteobacteria* at the phyla level as well as higher *Parabacteroides* at the genera level were observed in the Δ*saaS* group (Figure 4f). Furthermore, *Firmicutes*, *Patescibacteria* and *Actinobacteriota* had lower relative abundances in the Δ*saaS* group when compared with the WT group. At the genus level, four additional genera were identified in colon contents. In comparison to the WT group, the Δ*saaS* group had a greater presence of *Muribaculaceae_norank, Lachnospiraceae_uncultured* but lower abundances of *Ligilactobacillus* and *Mycobacterium*.

We further evaluated the microbial function change based on PICRUSt2 function prediction (Figure 4e-f). At 6 hpi, compared with WT infection, the Δ*saaS* infection decreased membrane transport and lipid metabolism but increased the microbial metabolism of cofactors and vitamins, cellular processes, as well as signaling, immune system and digestive system. This effect was further seen at 72 h with five more functions altered: xenobiotics biodegradation and metabolism were reduced, while nucleotide metabolism, folding/sorting/degradation, transport/catabolism and excretory system were heightened. These findings demonstrate that SaaS promotes the ability of *Salmonella* to modify gut microbiota composition over time as well as its functionality.

### The disruption of *Salmonella* to intestinal barrier by SaaS was associated with bacterial burden and inflammatory response

**Figure 5.**
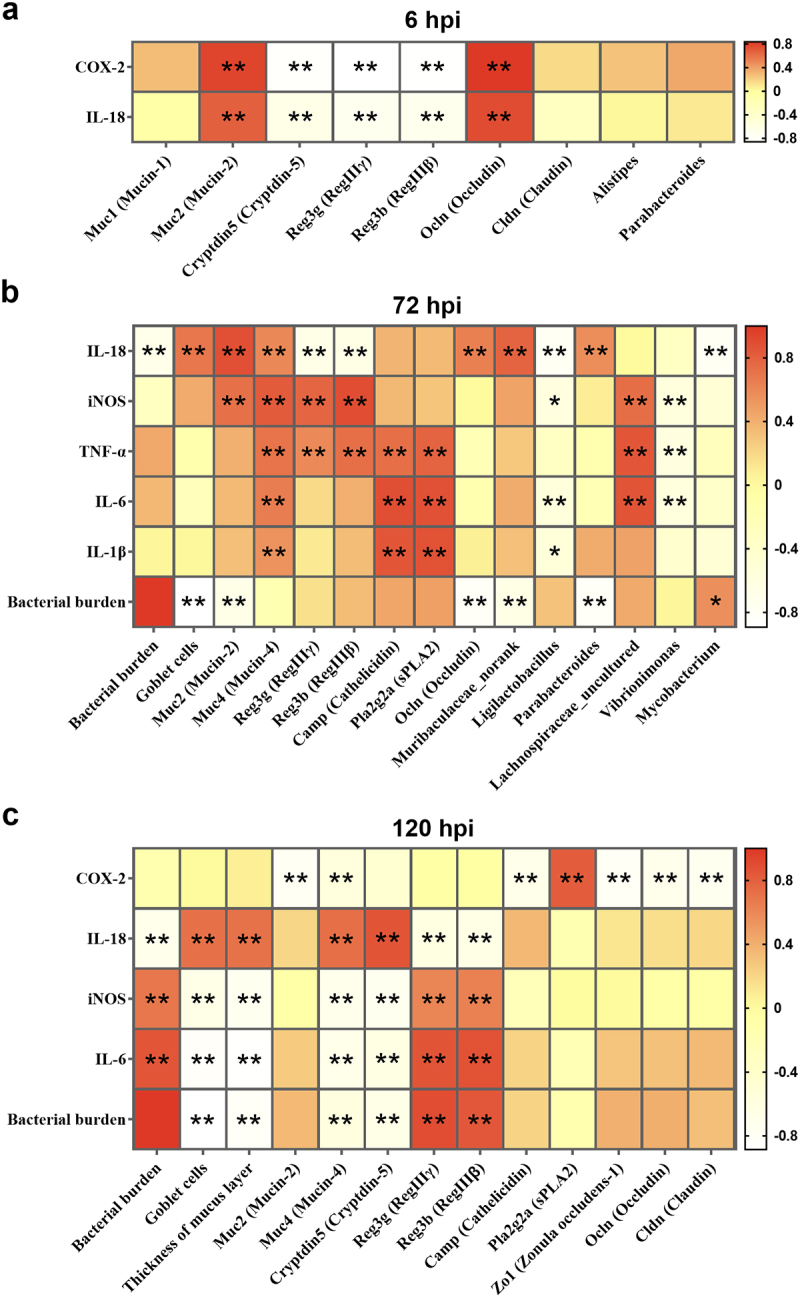
Altered inflammation levels exhibiting correlations with bacterial burden, intestinal barriers. (a) 6 hpi; (b) 72 hpi; (c) 120 hpi. Red color represents significant positive correlation and white color represents significant negative correlation, and the independent right color bars depict correlation coefficients. hpi: hours after infection. Correlation was considered significant when the absolute value of Spearman’s rank correlation coefficient (Spearman’s r) was > 0.5. *P < 0.05; **P < 0.01.

To evaluate potential relationships between inflammatory response and bacterial burden with intestinal barriers, Spearman’s correlation analysis was conducted (Figure 5). In general, IL-18 displayed the most intricate and powerful relationship with intestinal barriers, which was positively correlated with *Muc2* and *Ocln* at 6 and 72 hpi, positively correlated with goblet cells and *Muc4* at 72 and 120 hpi, but negatively correlated with *R3g3g* and *Reg3b* all through the entire period. *Il18* was also positively correlated with *Muribaculaceae_norank* and *Parabacteroides* but negatively correlated with *Ligilactobacillus and Mycobacterium* at 72 hpi. Following IL-18, COX-2 displayed the distinctive relationship with intestinal barriers. Different from those at 6 hpi, COX-2 showed the strongest negative correlation with intestinal barriers like *Muc2/4* and *Camp* in mucosal barrier and all markers in mechanical barrier.

Except IL-18 and COX-2, the correlation between other cytokines and barriers was regular and clear. At 72 hpi, inflammatory response was positively correlated with all mucosal barrier indicators. For biological barrier, inflammatory response was found to be positively associated with *Lachnospiraceae_uncultured* but negatively associated with *Vibrionimonas* plus *Ligilactobacillus*. At 120 hpi, inflammatory response was negatively correlated with goblet cells, thickness of mucus layer, *Muc4* and *Cryptdin5* but positively correlated with *Reg3g* and *R3g3b*, presenting a totally opposite trend with IL-18. Notably, *Muc4* showed the strongest correlation with all cytokines throughout the entire period, which suggests it may be a potential target in SaaS-mediated *Salmonella* invasion.

For bacterial burden, a steady negative correlation with *Il18* and goblet cells was observed at both 72 and 120 hpi. At 72 hpi, bacterial burden displayed a positive association with *Mycobacterium*, but displayed a negative association with *Muc2*, *Ocln*, *Muribaculaceae_norank* and *Parabacteroides*. At 120 hpi, bacterial burden displayed a positive association with IL-6, iNOS and *Reg3* family, but displayed a negative association with thickness of mucus layer, *Muc2* and *Cryptdin5*. These findings confirmed that SaaS helps *Salmonella* to disrupt intestinal barriers by mediating inflammatory responses.

### SaaS affects MAPK signaling pathway by mediating the activation pathway

**Figure 6.**
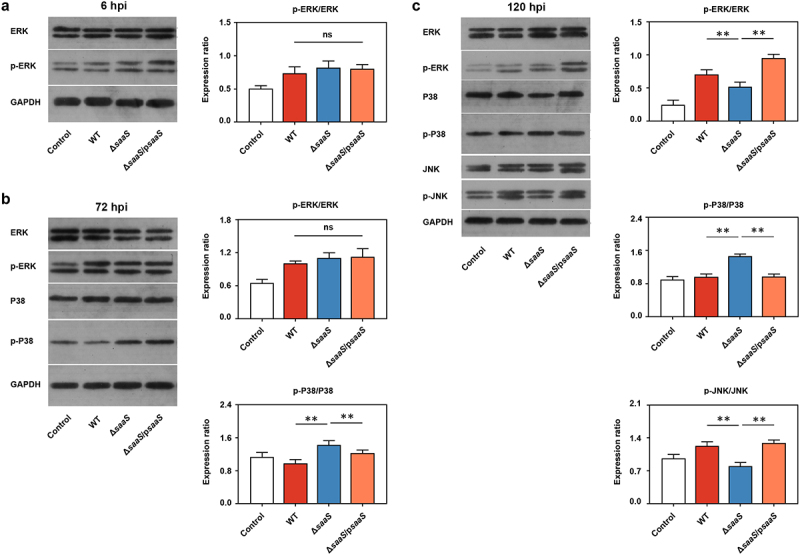
Effects of SaaS on protein expressions in MAPK signaling pathways. (a) Phosphorylation level of ERK in western blotting analysis at 6 hpi; (b) Phosphorylation level of ERK and P38 in western blotting analysis at 72 hpi; (c) Phosphorylation level of ERK, P38 and JNK in western blotting analysis at 120 hpi. hpi: hours after infection. Data are represented as means±SD from three independent experiments. Statistical significance was assessed using Student’s *t*-test. *P < 0.05, **P < 0.01.

The expression of pro-inflammatory molecules such as iNOS, COX-2, IL-1β, IL-6, IL-18 and TNF-α is known to be regulated by NF-*k*B (Nuclear factor kappa beta) and MAPK (Mitogen-activated protein kinase) signaling pathways. In general, it is not just the transcriptional activation of target genes but also the total and phosphorylation levels of proteins that are essential for the functioning of MAPK signaling pathway.^33^ According to mRNA expressions of *P38*, *Erk1/2* (encoding extracellular signal regulated kinase 1 and 2) and *Jnk1/2* (encoding c-Jun N-terminal kinase 1 and 2) (Fig S2), their corresponding protein levels were examined in order to evaluate both pathways’ roles in SaaS mediated *Salmonella* pathogenicity (Figure 6 and Fig S2). For ERK-MAPK route, there was a continuous increase in phosphorylation levels between all groups (Figure 6a-b), with a significant difference (*P* < 0.01) being observed between WT group and ΔsaaS group at 120 hpi (Figure 6c). This suggests an activation process for this particular pathway. On the other hand, P38-MAPK route was earlier shown to be regulated by SaaS due to the higher phosphorylation expression found at 72 hpi in the ΔsaaS group than the WT group, which aligned with the anti-inflammatory phenotype at 72 hpi (Figure 6b-c). Finally, the JNK-MAPK route was activated by SaaS. As shown in Figure 6c and Fig S2, both the mRNA and phosphorylation levels were significantly higher (*P* < 0.01) in the WT group than in the Δ*saaS* group at 120 hpi. Overall, even though suppression of P38-MAPK route continued, both JNK- and ERK-MAPK routes were subsequently augmented, resulting in a pro-inflammatory phenotype at 120 hpi. In regards to NF-kB pathway, there was no significant difference in both mRNA and protein levels of NF-kB among all groups during entire infection period (Fig S2d). These results revealed that it’s MAPK rather than NF-kB pathway which gets induced by SaaS during *Salmonella* infection.

**Figure 7.**
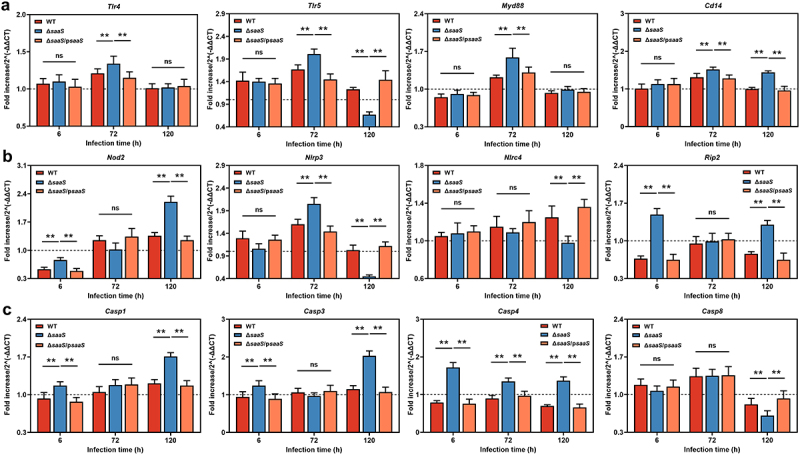
Effects of SaaS on the activation pathway of MAPK in colon. (a) Expressions of toll-like receptor systems including *Tlr4*, *Tlr5*, *Myd88* and *Cd14* mRNA; (b) Expressions of nod-like receptor systems including *Nod2*, *Nlrp3*, *Nlrc4* and *Rip2* mRNA; (c) Expressions of caspase family including *Casp1*, *Casp3*, *Casp4* and *Casp8* mRNA. hpi: hours after infection. For mRNA expression, the control was set to 1 and indicated by dashed line. Data are represented as means±SD; n = 7–10. Statistical significance was assessed using Student’s *t*-test. *P < 0.05, **P < 0.01.

We further looked into how SaaS mediated MAPK pathway *in vivo*. Pattern recognition receptors (PRRs), such as Toll-like receptors (TLRs) and Nod-like receptors (NLRs), respond to stimuli from local or foreign pathogenic substances.^34,35^ TLRs and NLRs are two major forms of innate immune sensors, which give immediate responses against invading pathogens or tissue damage. The mRNA levels of TLRs (*Tlr4* and *Tlr5*), NLRs (*Nod2* encoding nucleotide-binding oligomerization domain 2, *Nlrp3* encoding NLR thermal protein domain associated protein 3 and *Nlrc4* encoding NLR family CARD domain-containing protein 4) and their related ligands or downstream factors including *Myd88* (encoding myeloid differentiation primary response gene 88), *Cd14* (encoding cluster of differentiation 14), *Rip2* (encoding receptor-interacting protein 2) and *Casp1/3/4/8* (encoding Caspase 1, 3, 4 and 8) were analyzed in mice colon infected with *S*. Enteritidis (Figure 7). The results showed that the expression trends for these factors matched the corresponding protein levels at infection time points. At 6 hpi, compared to those in the Δ*saaS* group, all factors, except not significant *Tlr4*, *Tlr5*, *Nlrp3* and *Nlrc4*, had significantly lower expressions in the WT group. Similarly, lower mRNA levels of *Tlr4*, *Tlr5*, *Nlrp3* and *Casp4* were observed at 72 hpi in WT-infected mice when compared to Δ*saaS*-infected mice. On contrary, higher mRNA levels of *Tlr5*, *Nlrp3*, *Nlrc4* and *Casp8* were found at 120 hpi in WT-infected mice compared to those in Δ*saaS*-infected ones. These findings suggest that through SaaS, *Salmonella* activates MAPK signaling pathway sequentially by mediating activation pathways.

## Discussions

*Salmonella* are important enteric pathogens that invade humans and animals, with *S*. Enteritidis being one of the most common strains. In order to colonize various tissue sites for further damage, *Salmonella* needs to quickly adapt to different environments *in vivo* and break through the complex intestinal barrier. Recent research has found that sRNAs can regulate various genes post-transcriptionally which allows bacteria like *Salmonella* to adjust accordingly. SaaS, a novel sRNA, was identified as regulating expressions of virulence genes such as *invA*, *prgJ* and *ssa* operon in a simulated intestinal environment.^21^ Among them, the decrease in expression of *invA* and *prgJ* which are part of SPI-1 could weaken the initial invasion and survival of Δ*saaS*; this explains why there was a reduction at 72 hpi. When conditions changed from invasion to replication, *ssa* operon (*ssaV*, *ssaR*, *ssaT*, *ssaQ*, and *ssaU*) along with *spiA* belonging to SPI-2 type three secretion system (T3SS), like a baton, played their roles. The combination of increased expression of *spiA*, *ssaQ*, and *ssaU* as well as decreased levels of *ssaV*, *ssaR* and *ssaT* may have been responsible for the faster rate of replication seen at 120 hpi in this work but subsequently limited and delayed death that reported before.^21^ Further research into how SaaS interacts with virulence factors will be beneficial in understanding its role in pathogenesis better. At the primary period, *Salmonella* was observed to have a general anti-inflammatory effect, which was supported by the inhibition of virulence factors like *spvC* and AvrA.^14,36^ This enabled it to escape from immune response for better survival. Following this, onset of a potential cytokine storm could be observed based on the high levels of key pro-inflammatory cytokines iNOS, COX-2 and IL-6. Death caused by cytokine storms is the primary cause of mortality in animals infected with *Salmonella*.^37,38^ It was observed that iNOS and COX-2 are typically expressed together, indicating an extremely terrible condition.^39,40^ This suggests that SaaS modulates host immune response by regulating intestinal inflammation over time. However, how exactly increased colonization happens and regulated inflammation contributes toward damage caused by *Salmonella* remains unclear.

The goblet cells and the secretions they produce, such as MUC1/2/4 and AMPs, act as a first line of defense against *Salmonella*.^26,41,42^ Although the mucus layer does not appear to be impaired during initial infection, research suggests that bacteria can still penetrate intestinal regions with an intact mucus barrier,^43,44^ which was consistent with the death of goblet cells in this work. Though SopB wasn’t involved in the SaaS-mediated death of goblet cells, its roles in helping *Salmonella* maintain its concrete SCVs^45^, regulate inflammasome activation^46^, and promote *Salmonella* survival in B cells^47^ provide additional evidence that SaaS enhances *Salmonella* virulence. A novel and unknown mechanism of SaaS-mediated death of goblet cells was worth being investigated. The decreased *Muc1/2* levels also confirmed that *Salmonella* can penetrate an intact mucus barrier, and SaaS facilitates this invasion, thus triggering a heightened defensive response such as more AMPs being released. Continuously promoted invasion, in turn, weakened this defensive response^48^ and caused more harm to the mucosal barrier at later infection. The damage was further exacerbated by a decrease in IL-18, an interleukin responsible for antiapoptosis and mucosal restitution.^49,50^ At this point, it is noteworthy that AMPs like RegIII and sPLA2 also have TLRs-mediated pro-inflammatory properties.^48^ It was speculated that mediated by SaaS, *Salmonella* affected TLR 4/5 levels and then regulate RegIIIβ/γ secretion, thereby modulating the inflammatory response and compromising the innate defense.^51^ Decreased expression of *Cldn* at 72 hpi in this work was also confirmed by studies involving conventional intestine infections with *Salmonella*.^10^ In fact, the expression of *Cldn* can vary according to factors such as the type of injury, time of onset, and duration of the inflammatory response.^52^ For the slight increase at the beginning of infection, the body’s attempts to seal the intestinal barrier in order to prevent excessive electrolyte loss via diarrhea could be the possible reason.^53^ Furthermore, increased expression of *Cldn* has been associated with inflammation or inflammatory bowel diseases according to multiple reports,^54^ which supported our observation at 120 hpi. *Salmonella* achieves these diverse regulations usually by controlling TJs distribution or damaging interaction between them using effector proteins.^14,55^ As the largest component of T3SS, SsaV proteins are promoted by SaaS through direct combination with *ssaV* mRNA.^21^ This promotion further intensifies injection of effector proteins, resulting in the increased uptake of *Salmonella* into the host cells and initiated inflammation which disrupts gut microbiota.^56^ Some beneficial microbiota, such as *Alistipes*, *Parabacteroides* and *Lachnospiraceae_uncultured*, were shown to have decreased relative abundance here. *Alistipes* has been associated with the recovery of colitis. Decreased relative abundance of *Alistipes* was seen alongside inflamed gut environments and decreased *Muc1* and *Cldn* expressions just like this study,^57–59^ suggesting a synergistic effect between intestinal barriers for successful depletion mediated by SaaS. Also, the depletion of *Parabacteroides* and *Lachnospiraceae_uncultured* was confirmed by multiple *Salmonella* infection models, especially those involving higher virulence *Salmonella* infection.^56,60^ While for *Ligilactobacillus*, a probiotic bacteria used to treat enteric infections,^61^ the increased abundance could be utilized by *Salmonella* to suppress inflammatory response for immune escape as reported here,^62^ confirming the negative correlation between it and pro-inflammatory factors. On the other hand, SaaS increased the relative abundance of deleterious bacteria or making general bacteria harmful such as the *Mycobacterium*. As an interesting concept “like will to like”,^63^ the increased *Salmonella* might be responsible for the increased abundance of *Proteobacteria* because it includes the similar species such as *Enterobacteriaceae* family, thus increasing susceptibility to infection.^64^ The diversity and associated bioactivity of gut microbiota have important effects on the host’s metabolic homeostasis and immune system.^65–67^ Therefore, a decrease in microbial diversity could be responsible for the malfunctioning metabolism and immune system seen here.

MAPK is critical intersection pathway of cell proliferation, stress responses, inflammation, apoptosis and other signal transduction pathways.^32,68^ The MAPK pathway has been shown to be time-dependently regulated by SaaS-mediated *Salmonella*, which is in agreement with the inflammatory response. As the starter of MAPK inflammatory signaling cascades, NLRs and TLRs systems were both suppressed during early infection but enhanced at a later stage, correlating with the decrease of P38 activity and increase in ERK and JNK activities. This suggests a direct regulatory cascade between them. Although NLRs and TLRs usually participate in host defense and tissue repair,^69^ they have also been utilized by T3SS, such as TcpS mediating TLR^70^ and PrgJ interacting with NLRC4 inflammasome,^71,72^ to enhance the virulence of *Salmonella in vivo*. Inflammasome is innate immune defense mechanism that can become overactivated, leading to the death of host. Both inflammasomes *Nlrp3* and *Nlrc4* were activated by SaaS, with *prgJ* being predicted as a target mRNA of SaaS, which was found to be positively regulated by it,^21^ suggesting activation of NLRC4 through interaction between SaaS and *prgJ*. Furthermore, an induced NLRC4-Caspase 8 axis based on inhibition of Caspase 1 was observed here, which was confirmed via an apoptosis model built in recent years.^73,74^ These details raise the possibility that SaaS may interact with *prgJ* to activate the NLRC4 inflammasome, resulting in NLRC4-Caspase 8-mediated apoptosis of cells. Meanwhile, researches showing that inhibiting JNK signaling can greatly dampen apoptosis in epithelial cells that infected with *Salmonella* ^75,76^. Correspondingly, the JNK signaling of MAPK was stimulated in this work, which implies an increase in apoptosis mediated by SaaS.

## Conclusion

This study is the first to investigate in-depth how *Salmonella* sRNA affects the intestine, and it revealed a novel regulation of sRNA on MAPK signaling pathway. The present study demonstrates that SaaS promotes the bacterial colonization by increasing itself expression and damaging the physical and mucus barriers. Meanwhile, SaaS damaged intestinal homeostasis and function by decreasing levels of beneficial bacteria and increasing levels of harmful bacteria. Furthermore, SaaS initially alleviates intestinal inflammation by suppressing P38 MAPK pathway and its activation to promote survival and colonization, but it eventually promotes intestinal inflammation by intensifying JNK and ERK MAPK pathways and its activation, raising a cytokine storm. Our research has potential significance as it provides new paradigms for interactions between sRNA and host intestinal immune response during infection by *Salmonella* or other pathogens.

## Materials and methods

### Ethics statement

All experiments were carried out in compliance with the relevant guidelines and regulations of the Ethical Committee of Experimental Animal Center of Nanjing Agricultural University.

### Construction of SaaS deletion mutant and complemented strain

*S*. Enteritidis strain NCM61 (Reference genome CP032851.1) isolated from meat-contact surfaces was used as the wild-type (WT).^77^ The SaaS-deletion mutant Δ*saaS* and complemented strain Δ*saaS*/p*saaS* were obtained from the same source as indicated in our previous work.^21^ Briefly, the Δ*saaS* was constructed based on WT according to allelic exchange using the suicide plasmid. First, a bridged target fragment Δ*saaS*::Kn (upstream homologous arm-Knr gene-downstream homologous arm) was constructed and subcloned into the suicide plasmid pCVD442, obtaining the pCVD442-Δ*saaS*::Kn. Secondly, the donor strain β2155/pCVD442-Δ*saaS*::Kn was obtained by transferring pCVD442-Δ*saaS*::Kn into *Escherichia coli* β2155. The conjugation test of above donor strain and the recipient strain WT was performed, and the *Salmonella* clones obtaining Knr were collected and named as Sen/pCVD442-Δ*saaS*::Kn. Finally, deletion mutants (Δ*saaS*) were obtained by electroporating plasmid pCP20 into the competent cells of Sen/pCVD442-Δ*saaS*::Kn and named as *S*. Enteritidis strain NCM282.

Δ*saaS*/p*saaS* was then constructed based on Δ*saaS* using the low-copy pRK415 expression vector, which was reported in Keen et al., 1988.^78^ Similarly, the *saaS* gene and pRK415 expression vector were amplified, and the digested products were ligated for 2 h at 37°C to yield the constructed plasmid pRK415-*saaS*. pRK415-*saaS* was subsequently transferred into Δ*saaS* and selected on tetracycline (Tc, 10 μg/mL) plates to yield the complemented strain (Δ*saaS*/p*saaS*).

### Bacterial cultures and growth conditions

*S*. Enteritidis strain WT, mutant (Δ*saaS*) and complement (Δ*saaS*/p*saaS*) were streaked and grown twice on Luria-Bertani agar (LB; HopeBiotechnology, China) overnight for single colony isolation and then incubated in 6 mL of fresh LB broth at 37°C for 20 h. The cells were harvested by centrifugation at 8000×g for 5 min at 4°C and rinsed three times with 0.85% NaCl solution without activating the cold shock proteins CspE and CspD (Fig S3). Subsequently, the pellets were resuspended in 0.85% NaCl solution to a final concentration of approximately 10^9^ CFU/mL by measuring the OD_600_ for subsequent experiments.

### Animal experiment

Specific-pathogen-free (SPF) female BALB/c mice aged 6 weeks were housed under SPF conditions (humidity, 60 ± 10%; light cycle, 12 h/12 h; temperature, 23.0 ± 0.5°C) (SYXK<Jiangsu>2011–0037). The mice had free access to their diet and water. Following adaptation to the new environment for 1 week, mice were randomly divided into four groups, including PBS (control), WT, Δ*saaS* and Δ*saaS*/p*saaS*-infected groups; 1 × 10^8^ CFU *S*. Enteritidis strains under exponential phase, respectively, in 100 µL of PBS were used for oral gavage to mice that were fasted for 4 h before infection. At 6-, 72- and 120- hour post-infection (hpi), the colon, colonic content and cecum were collected and snap-frozen, separately.

### Bacterial burden

The colon and cecum were homogenized and plated on XLD agar. CFU was normalized to the weight of each sample.

### ELISA assays

Frozen colon (40–50 mg) was lysed in protein extraction buffer containing protease and phosphatase inhibitors (Beyotime, China). The samples were centrifuged at 13,000 g for 5 min at 4°C, and the protein concentration in the supernatant was determined using a BCA protein assay kit (Thermo Scientific, USA). The IL-1β, IL-6, TNF-α, iNOS and sIgA levels were assessed by ELISA (Angle gene, China) at 6, 72 and 120 hpi according to the kit instructions.

### Histological observations

The colon samples were fixed in Carnoy’s fixative solution for alcian blue-periodic acid Schiff (AB-PAS) and 4% paraformaldehyde for hematoxylin and eosin (H&E). The samples were processed with routine histological procedures, dehydration, paraffin embedding, section cutting, deparaffinization and stained according to the previous study.^78^ The relative number of goblet cells was counted on ten random points per section. The mucus thickness was determined by measuring 10 randomly chosen points per section. These images were obtained from each individual by an operator unaware of sample status using Olympus B×51 (Olympus, Tokyo, Japan).

### Quantitative real-time PCR

The colon (10–20 mg) samples were collected, and total RNA was isolated with FastPure Cell/Tissue Total RNA Isolation Kit (Vazyme, China). Reverse transcription of 800 ng of RNA was then performed using a HiScript III RT SuperMix for qPCR (+gDNA wiper) (Vazyme), and 1 µL of cDNA were used for real-time reaction using ChamQ Universal SYBR qPCR Master Mix (Vazyme). A QuantStudio 6 Flex system (Applied Biosystems, United States) was applied with the following protocol: initial denaturation at 95°C/30 s; 40 cycles of denaturation at 95°C/5 s and annealing at 60°C/34 s; and a final melting curve program of 15 s at 95°C, 1 min at 60°C, and 15 s at 95°C. Primers were designed using Primer Premiere (Version 5.0; San Francisco, CA, USA), synthesized (GenScript, China) and listed in Table S1. In parallel, *Gapdh* and *Actb* were included as dual internal references for normalization of the gene expression. Fold changes in gene expression were calculated using the 2^−ΔΔCT^ method, where ΔΔCT = ΔCT (WT or Δ*saaS* or Δ*saaS*/p*saaS*) - ΔCT (control), ΔCT = CT (target gene) - CT (geometric average of *Gapdh* and *Actb*).^79^

To compare the expression of SaaS in colonized *Salmonella* in colon and cecum, bacteria were collected and rinsed with PBS three times. Total RNA was extracted with a simple total RNA extraction kit (Tiangen, China) following the manufacturer’s protocol. The process of synthesizing cDNA and determining SaaS expression levels was carried out using the same method as mentioned above. 16S rRNA was used as an internal reference to normalize gene expression data. Fold changes in gene expression were calculated using the 2^−ΔΔCT^ method, where ΔΔCT = ΔCT (Treatment group) - ΔCT (cecum at 6 hpi), ΔCT = CT (SaaS) - CT (16S rRNA). Treatment group means the cecum at 72 hpi or cecum at 120 hpi or colon at 6 hpi or colon at 72 hpi or colon at 120 hpi.

To evaluate the half-life of SaaS, rifampicin stability assays were carried out. Test cultures were cultured overnight at 37°C to exponential phase, adding rifampicin to a final concentration of 250 μg/mL. At 0, 5, 10, 15, and 20 min, the cells were quickly harvested by centrifugation at 12,000×g for 2 min. Pellets were frozen in liquid nitrogen and stored at −80°C. The process of extracting RNA, synthesizing cDNA and determining SaaS expression levels was carried out using the same method as mentioned above. 16S rRNA was used as an internal reference to normalize gene expression data. Fold changes in gene expression were calculated using the 2^−ΔΔCT^ method, where ΔΔCT = ΔCT (Treatment group) - ΔCT (0 min), ΔCT = CT (SaaS) - CT (16S rRNA). Treatment group means the 5 min or 10 min or 15 min or 20 min.

### Cell experiments

#### Bacterial invasion and survival assays

Before infection, Caco-2 cells were washed three times with prewarmed PBS, and the medium was replaced with fresh DMEM without antibiotics and fetal bovine serum. Monolayers of cells were infected with an exponential phase bacterial culture (MOI = 10), then incubated for 3 h at 37°C in a 5% CO_2_ atmosphere. After incubation, the wells were washed six times with PBS to remove unattached bacteria. Caco-2 cells were then disrupted with 1% Triton X-100 at 37°C for 5 min. Lysate dilutions were plated on XLD agar, and the attachment efficiency was determined by counting the colony-forming units (CFU) per milliliter. The 100 and 10 μg/mL gentamicin were then used to kill surface and extracellular adhered bacteria. The survival levels at 18 h were determined by comparing bacterial recovery from the initial inoculum.

#### Cell cytotoxicity assays

To verify the viability of Caco-2 cells during infection, the supernatants from above tests were collected, and the release of lactate dehydrogenase (LDH) was determined according to the instruction of lactate dehydrogenase assay kit (Jiancheng, China). The relative LDH release was calculated as LDH release in the supernatant/(LDH release in supernatant + LDH release in cell fraction)×100%.

### S rRNA gene sequencing

We detected the composition of intestinal microflora in the colon of mice in the control and *S*. Enteritidis-infected groups. Total DNA was extracted from colonic contents using the E.Z.N.A.® Soil DNA Kit (Omega Biotek, USA) according to manufacturer’s protocols. The V3-V4 hyper-variable regions of the bacteria 16S rRNA gene were targeted and selected for PCR amplification using a barcode primer 515F (5’-GTGCCAGCMGCCGCGG-3’) and 806 R (5’-GGACTACHVGGGTWTCTAAT-3’) in which barcode is an eight-base sequence unique to each sample. The subsequent detailed PCR protocol and methods for all analyses can be found in Supplementary Material.

### Correlation analysis

The Spearman’s correlation coefficients were assessed to determine the relationships between inflammation levels/bacterial burden and intestinal barrier. For each time point, the indicator with no significant difference between treatment groups was excluded. To compare two random indicators, the data from four groups in each indicator was utilized in this comparison method. Correlation was considered significant when the absolute value of Spearman’s rank correlation coefficient was greater than 0.5 and *P*-value was smaller than 0.05.

### Western blotting

Frozen colon (50 mg) was lysed in protein extraction buffer containing protease and phosphatase inhibitors (Beyotime). The samples were centrifuged at 13,000 g for 10 min at 4°C, and the protein concentration in the supernatant was determined using a BCA protein assay kit (Thermo Scientific). Protein (60 μg) was separated by sodium dodecyl sulfatepolyacrylamide gel electrophoresis (SDS-PAGE) and transferred onto polyvinylidene fluoride (PVDF; Millipore, Billerica, USA) membranes. All the primary antibodies were from Cell Signalling Technology (CST, USA) and used with the suitable dilution ratio of 1:1000 for ERK, p-ERK, P38, p-P38, JNK and GAPDH and 1:2000 for p-JNK. Membranes were incubated with appropriate secondary antibodies Goat anti-Mouse IgG (H+L) or Goat anti-Rabbit IgG (H+L) (Thermo Scientific; 1:5000). Quantification of the protein bands was performed with ImageJ (Version 1.53c; NIH, Bethesda, MD, USA) and normalized to GADPH.

### Statistical analysis

SAS software (Version 9.2; SAS Institute Inc., USA) was used for statistical analysis. The differences between two groups were analyzed using Student’s *t*-test. Values of *P* ≤ 0.05 or 0.01 were considered to be statistically significant or highly significant, respectively. Data were expressed as means ± standard deviation (SD) and figures were constructed using the GraphPad Prism (Version 5.0.3; GraphPad Software Inc., USA). More details about the materials and methods are seen in Supplementary Material.

## Supplementary Material

Supplemental Material

## Data Availability

The data that support the findings of this study are available in figshare at https://figshare.com/s/cfe1f4b520b47dd62967.
