# Supplementary material for: SaaS sRNA promotes Salmonella intestinal invasion via modulating MAPK inflammatory pathway
Source: Gut Microbes. 2023 May 9;15(1):2211184. doi: 10.1080/19490976.2023.2211184 (PMC10171124; doi:10.1080/19490976.2023.2211184)
Supplement: Supplemental Material [file KGMI_A_2211184_SM8868.docx]

SaaS sRNA promotes *Salmonella* intestinal invasion via modulating MAPK inflammatory pathway

Linlin Cai, Yunting Xie, Liangting Shao, Haijing Hu, Xinglian Xu*, Huhu Wang* and Guanghong Zhou

Jiangsu Collaborative Innovation Center of Meat Production and Processing, Quality and Safety Control, Nanjing Agricultural University, Nanjing 210095, P.R. China

Corresponding author, E-mail address: xlxus@njau.edu.cn (Xinglian Xu), huuwang@njau.edu.cn (Huhu Wang).

# Materials and methods

***Expression of cspD, cspE and sopB mRNAs***

The bacteria were collected and washed with PBS for three times at room temperature (RT) or 4℃. The process of extracting RNA, synthesizing cDNA and determining SaaS expression levels was carried out using the same method as mentioned in the text. 16S rRNA was used as an internal reference to normalize gene expression data. Fold changes in gene expression were calculated using the 2^−ΔΔCT^ method, where ΔΔCT = ΔCT (Treatment group) **-** ΔCT (WT at RT), ΔCT = CT (*cspD* or *cspE*) **-** CT (16S rRNA). Treatment group means the Δ*saaS* at RT or Δ*saaS/*p*SaaS* at RT or WT at 4℃ or Δ*saaS* at 4℃ or Δ*saaS/*p*SaaS* at 4℃, while for *sopB* mRNA, the ΔΔCT = ΔCT (Δ*saaS* or Δ*saaS/*p*SaaS*) **-** ΔCT (WT), ΔCT = CT (*sopB*) **-** CT (16S rRNA).

## Cell experiments

### Bacterial invasion and survival assays

Bacterial invasion and survival assays were performed as described method^1^ for Caco-2 cells. Before infection, Caco-2 cells were washed three times with prewarmed PBS, and the medium was replaced with fresh DMEM without antibiotics and fetal bovine serum. Monolayers of cells were infected with an exponential phase bacterial culture (MOI=10), then incubated for 3 h at 37°C in a 5% CO_2_ atmosphere. After incubation, the wells were washed six times with PBS to remove unattached bacteria. Caco-2 cells were then disrupted with 1% Triton X-100 at 37°C for 5 min. Lysate dilutions were plated on XLD agar, and the attachment efficiency was determined by counting the colony-forming units (CFU) per milliliter. The 100 and 10 μg/mL gentamicin were then used to kill surface and extracellular adhered bacteria. The survival levels at 18h was determined by comparing bacterial recovery from the initial inoculum.

### Cell cytotoxicity assays

To verify the viability of Caco-2 cells during infection, the release of lactate dehydrogenase (LDH) was determined according to the instruction of lactate dehydrogenase assay kit (Jiancheng, China). The supernatants from above tests were collected and frozen in micro-tubes at -80°C. Briefly for the test, 20 μL of the supernatant were transferred to 96-well polystyrene plates. Subsequently, 30 μL of the reaction mixture from the LDH detection kit were added and incubated for 15 min at 37°C, then 25 μL of 2, 4-dinitrophenylhydrazine were added followed by another 15-min incubation. Finally, 250 μL of 0.4 mol/L NaOH were transferred to plates. The contents were mixed briefly on an orbital shaker and incubated for 5 min at room temperature, followed by the recording using a multimode microplate reader at 450 nm. The relative LDH release was calculated as LDH release in the supernatant /(LDH release in supernatant + LDH release in cell fraction)×100%.

## 16S rRNA gene sequencing

### DNA extraction and gene sequencing

We detected the composition of intestinal microflora in the colon of mice in the control and *S.* Enteritidis-infected groups. Total DNA was extracted from colonic contents using the E.Z.N.A.^®^ Soil DNA Kit (Omega Biotek, USA) according to manufacturer’s protocols. The V3-V4 hyper-variable regions of the bacteria 16S rRNA gene were targeted and selected for PCR amplification using a barcode primer 515F (5’-GTGCCAGCMGCCGCGG-3’) and 806R (5’-GGACTACHVGGGTWTCTAAT-3’) in which barcode is an eight-base sequence unique to each sample. The protocol was set as followed: 95°C/2 min, followed by 25 cycles at 95°C/30 s, 55°C/30 s, and 72°C/30 s and a final extension program at 72°C/5 min. PCR reactions were performed in 20 μL mixture containing 4 μL of 5 × FastPfu Buffer, 2 μL of 2.5 mM dNTPs, 0.8 μL of each primer (5 μM), 0.4 μL of FastPfu Polymerase, and 10 ng of template DNA. The amplified products were extracted from 2% agarose gels and purified with a AxyPrep DNA Gel Extraction Kit (Axygen Biosciences, USA) according to the manufacturer’s instructions.

As described in our previous study,^2^ purified PCR products were quantified by Qubit^®^ 3.0 (Life Technologies, [Carlsbad, CA, USA](https://www.bionity.com/en/companies/24328/life-technologies-corporation.html)). The Illumina Pair-End library was constructed by pooled DNA product following Illumina’s genomic DNA library preparation procedure. Then, the Illumina MiSeq platform was applied to pair-end (2×250) sequence amplicon library according to the standard protocols. Raw reads were demultiplexed and quality-filtered using QIIME (Version 1.17) with the following criteria. The 250-bp reads were truncated at any site receiving an average quality score <20 over a 10-bp sliding window, with the truncated reads that shorter than 50 bp discarded. Sequences were exactly matched and those overlapping longer than 10 bp were assembled.^3^ After that, the tags were clustered to operational taxonomic units (OTUs) with 100% similarity through Deblur denoising algorithm for detecting microbial changes at fine scale resolution.^4^ The phylogenetic affiliation of each 16S rRNA gene sequence was analyzed by uclust algorithm of USARCH (Version 11, http://www.drive5.com/usearch/manual/uclust_algo.html) against the SILVA (SSU138.1) 16S rRNA data with a confidence threshold of 80%.^3^

### Bioinformatics data analysis

According to the results of OTU analysis, the relative abundance of each OTU was defined at different taxonomic levels and diversity indices including community richness estimator (Chao1 and ACE), diversity indices (Shannon and Simpson). Principal coordinate analysis (PCoA) and clustering analysis (http://sekhon.berkeley.edu/stats/html/ hclust.html) were applied on the basis of the OTUs to offer an overview of the colonic microbial composition. The distance-based analysis of molecular variance (AMOVA) was conducted to further assess the significance between different group samples.

### Functional prediction of the microbial genes

The Phylogenetic Investigation of Communities by Reconstruction of Unobserved States (PICRUSt) program based on the the Kyoto Encyclopedia of Genes and Genomes (KEGG) database was used to predict the functional alteration of colon microbiota in different samples.^5^ The OTU data obtained were used to generate BIOM files formatted as input for PICRUSt v1.1.09 with the make.biom script usable in the Mothur. OTU abundances were mapped to Greengenes OTU IDs as input to speculate about the functional alteration of microbiota.

**Supplementary figure captions**

Fig S1. Effects of SaaS on expressions of *sopB* mRNA. Data are represented as means±SD. Statistical significance was determined using Student’s *t*-test. **P*<0.05, ***P*<0.01.

Fig S2. Effects of SaaS on NF-kB and MAPK signaling pathway. (a) Expressions of *P38* mRNA; (b) Expressions of *Erk1* and *Erk2* mRNAs; (c) Expressions of *Jnk1* and *Jnk2* mRNAs; (d) Expressions of *Nfkb* mRNA and protein levels. Data are represented as means±SD. Statistical significance was determined against Δ*saaS* using Student’s *t*-test. **P*<0.05, ***P*<0.01.

Fig S3. Effects of centrifugation temperature on expressions of *cspD* and *cspE* mRNAs. Data are represented as means±SD. Statistical significance was determined using Student’s *t*-test. **P*<0.05, ***P*<0.01.

Table S1. Primers used for RT-qPCR analysis.

| Gene | Primer-F (5’-3’) | Primer-R (5’-3’) | Products | Reference |
| --- | --- | --- | --- | --- |
| SaaS | TGTATCTGTCACTTAAGTAAAG | GCGTGTTAATGCGCAGTCTG | 73 bp | This study |
| *cspD* | GGTGGAATAATGGGCGAAA | GGAAACGGGTACTGTAAAGTGG | 97 bp | This study |
| *cspE* | GGCACCGTTAGTGATTTCG | GAAGATGGCAGCAAAGACG | 108 bp | This study |
| *sopB* | GGAATTGTAAAAGCGGCAAA | TTTTCTGTCCACCGCTATCC | 129 bp | (6) |
| 16S rRNA | CGGGGAGGAAGGTGTTGTG | GAGCCCGGGGATTTCACATC | 178 bp | (7) |
| *Il18* | GTTCCTGTAGAGCTTCATTCCCA | TGTACTCATCGTTGTGGGAACA | 108 bp | This study |
| *Cox2* | CAATGAGTACCGCAAACGC | TGGTCTCCCCAAAGATAGCA | 176 bp | This study |
| *Muc1* | TGCCCTTCCAAGTGAGGAAA | CTGGAGTGGTAGTCGATGCT | 93 bp | This study |
| *Muc2* | CACCATCTACCACGTCTTCCAA | CACCATCTACCACGTCTTCCAA | 158 bp | This study |
| *Muc4* | GCGAGGGATGTCAGGACTAA | TTGCCCTCTTTGCTGGTTTC | 110 bp | This study |
| *Zo1* | TGTTTATGCGGACGGTGGCG | TCCATTGCTGTGCTCTTAGCGG | 132 bp | This study |
| *Ocln* | TAAAAGGGCTCCCACGAAGG | ACGTCGTCTAGTTCTGCCTG | 225 bp | This study |
| *Cldn* | CTGTGGATGTCCTGCGTTTC | TTACCATCAAGGCTCGGGTT | 109 bp | This study |
| *B3gnt6* | CACACGGCCAACGTACTGA | CAGGCACAGAACCAACCATGA | 88 bp | (8) |
| *St6galnac1* | CTTTCTGCCCAACATCACTC | GTCATGGCTGTCTATCTCCC | 271 bp | This study |
| *Cryptdin1* | CTAGTCCTACTCTTTGCCCT | TTGCAGCCTCTTGATCTACA | 206 bp | (9) |
| *Cryptdin4* | TGGCCTCCAAAGGAGATAGACA | AGGCTGATCCTATCCAAAACACA | 94 bp | (10) |
| *Cryptdin5* | GTCCAGGCTGATCCTATCCA | GATTTCTGCAGGTCCCAAAA | 202 bp | (9) |
| *Reg3g* | AGCCACAAGCAAGATCCCAA | GGCCATAGTGCACACAGAGT | 138 bp | (11) |
| *Reg3b* | ACAGACAAGATGCTGCCTCC | GAGCCCTTGGGGCAACTAAT | 140 bp | (11) |
| *Camp* | CAGCCCTTTCGGTTCAAGAA | CCCACCTTTGCGGAGAAGT | 57 bp | (10) |
| *Pla2g2a* | ACAGGTCCAAGGGAACATTG | TCTGGTTTGCAGAACAGGTG | 257 bp | (12) |
| *Lyz* | ATGGCGAACACAATGTCAAA | GCGAGGAAGTGTGACCTCTC | 141 bp | (12) |
| *Tlr4* | GCCACCAGTTACAGATCGTC | GAGGCATCATCCTGGCATTT | 87 bp | This study |
| *Tlr5* | TAACTCACTGCTAGGGGGCT | TCCCCCGGAATGAAGTCTCT | 375 bp | This study |
| *Myd88* | GCCAGAGTGGAAAGCAGTG | TTGGGGCAGTAGCAGATAAA | 116 bp | This study |
| *Cd14* | GCAACGTGTCGTGGGCAACA | TGAGCGAGTGTGCTTGGGCA | 102 bp | This study |
| *Nod2* | GCCAGTACGAGTGTGAGGAG | CCCTGACGTGCTGTAGAAGG | 126 bp | This study |
| *Nlrp3* | GTGAAACAAAACGTGCCTTAGAA | GGAGGGCTTGATAGCAGTGAA | 170 bp | This study |
| *Nlrc4* | TCTCCAAGAGATGAAGTTGGTGG | GTTCCTGTAGAGCTTCATTCCCA | 149 bp | This study |
| *Rip2* | CCCTCTGCTTCATCATGACCTA | TCCTTCGGGTGCAGATTTGT | 142 bp | This study |
| *Casp1* | GGCTCACTTTTCATTGAGTCAC | ATCTGTAGCCTAAATTCTGGTTG | 122 bp | This study |
| *Casp3* | CGGAGCTGGACTGTGGCATT | GGATGAACCACGACCCGTCC | 153 bp | This study |
| *Casp4* | CCACATCACTTGTCCTACCGAGA | CCTTTTCAAATGATTGTTGCACCT | 136 bp | This study |
| *Casp8* | AACCGAGAGGAGATGGTGAGA | TCTTCCAGCTTACATTTGGGGA | 161 bp | This study |
| *Nfkb* | ATGGCAGACGATGATCCCTAC | TGTTGACAGTGGTATTTCTGGTG | 111 bp | This study |
| *P38* | ATGAGGAGATGACCGGATATGTG | GCAGCAGTTCAGCCATGATG | 126 bp | This study |
| *Erk1* | TCCTTTTGGATCTGGTCCTG | CCCCAGCAAAGTGAGAGAAG | 136 bp | This study |
| *Erk2* | GGAGCAGTATTATGACCCAAGTGA | TCGTCCACTCCATGTCAAACT | 81 bp | This study |
| *Jnk1* | GCAGAAGCAAACGTGACAACA | TTCTGAAATGGCCGGCTGAG | 184 bp | This study |
| *Jnk2* | GCAAAGAGAGCCTACCGTGAA | AGGACATTCTCTCATGGTCCAG | 190 bp | This study |
| *Gapdh* | TTTGGCATTGTGGAAGGGCT | GTCAGATCCACGACGGACAC | 236 bp | This study |
| *Actb* | TGAGCTGCGTTTTACACCCT | GCCTTCACCGTTCCAGTTTT | 198 bp | This study |


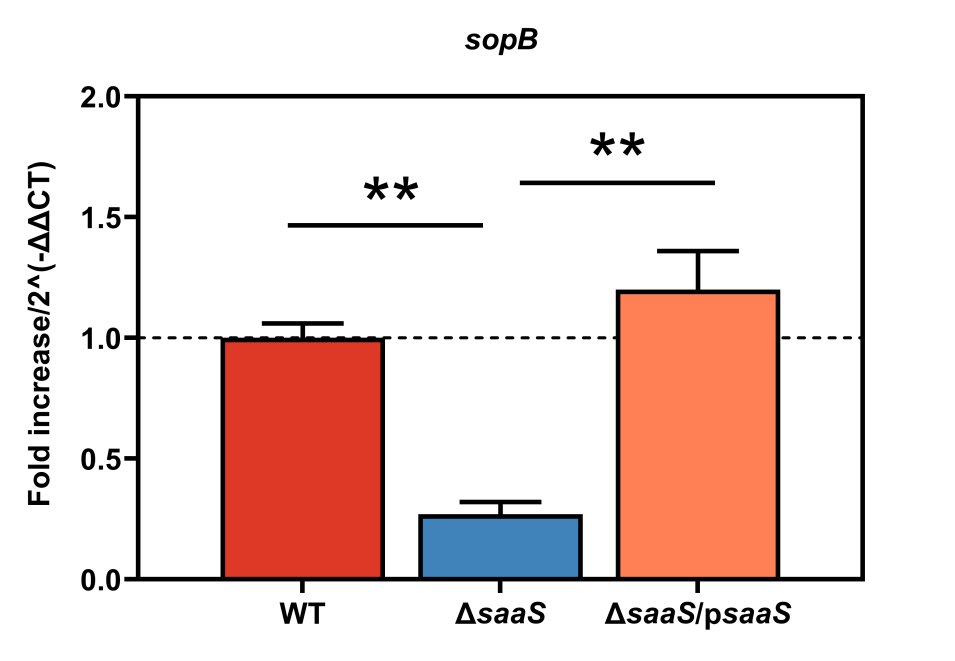


Fig S1


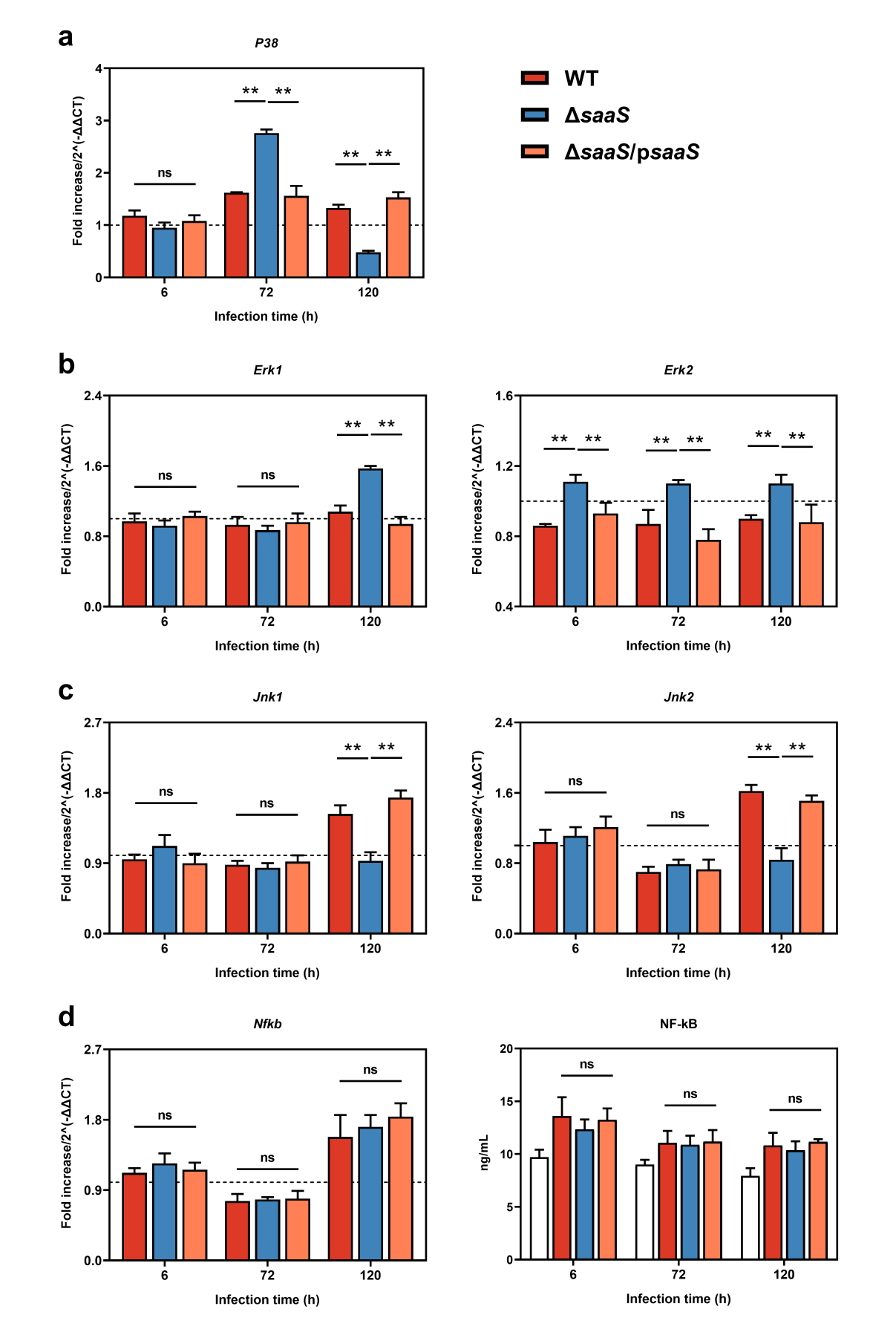


Fig S2


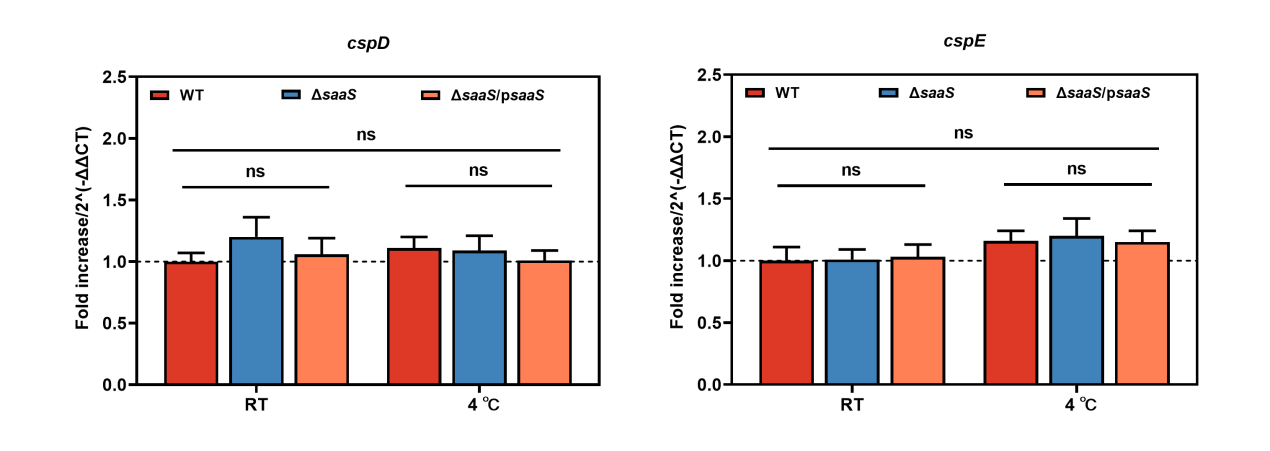


Fig S3

**References**

1. Yang B, Feng L, Wang F, Wang L. Enterohemorrhagic *Escherichia coli* senses low biotin status in the large intestine for colonization and infection. Nat Commun. 2015;6:6592-6605.

2. Xie Y, Wang C, Zhao D, Zhou G, Li C. Processing method altered mouse intestinal morphology and microbial composition by affecting digestion of meat proteins. Front Microbiol. 2020;11:511-523.

3. Caporaso JG, Kuczynski J, Stombaugh J, Bittinger K, Bushman FD, Costello EK, Fierer N, Peña AG, Goodrich JK, Gordon JI, et al. QIIME allows analysis of high-throughput community sequencing data. Nat Methods. 2010;7:335-336.

4. Cole JR, Wang Q, Cardenas E, Fish J, Chai B, Farris RJ, Kulam-Syed-Mohideen AS, McGarrell DM, Marsh T, Garrity GM, et al. The ribosomal database project: improved alignments and new tools for rRNA analysis. Nucleic Acids Res. 2009;37:141-145.

5. Langille MGI, Zaneveld J, Caporaso JG, McDonald D, Knights D, Reyes JA, Clemente JC, Burkepile DE, Vega Thurber RL, Knight R, et al. Predictive functional profiling of microbial communities using 16S rRNA marker gene sequences. Nat Biotechnol. 2013;31:814-821.

6. Hu GQ, Yang YJ, Qin XX, et al. *Salmonella* outer protein B suppresses colitis development via protecting cell from necroptosis[J]. Front Cell Infect Microbio, 2019, 9: 87-99.

7. Cai LL, Xie YT, Hu HJ, Xu XL, Wang HH, Zhou GH. A small RNA, SaaS, promotes *Salmonella* pathogenicity by regulating invasion, intracellular growth, and virulence factors. Microbiol Spectr. 2023;11:e0293822.

8. Wang XW, Spandidos A, Wang HJ, Seed B. PrimerBank: a PCR primer database for quantitative gene expression analysis, 2012 update. Nucleic Acids Res. 2012;40:1144-1153.

9. Inaba Y, Ashida T, Ito T, Ishikawa C, Tanabe H, Maemoto A, Watari J, Ayabe T, Mizukami Y, Fujiya M, et al. Expression of the antimicrobial peptide α-defensin/cryptdins in intestinal crypts decreases at the initial phase of intestinal inflammation in a model of inflammatory bowel disease, IL-10-deficient mice. Inflamm Bowel Dis. 2010;16:1488-1495.

10. Zhao Y, Chen F, Wu W, Sun M, Bilotta AJ, Yao S, Xiao Y, Huang X, Eaves-Pyles TD, Golovko G, et al. GPR43 mediates microbiota metabolite SCFA regulation of antimicrobial peptide expression in intestinal epithelial cells via activation of mTOR and STAT3. Mucosal Immunol. 2018;11:752-762.

11. Udomsopagit T, Miwa A, Seki M, Shimbori E, Kadota Y, Tochio T, Sonoyama K. Intestinal microbiota transplantation reveals the role of microbiota in dietary regulation of RegIIIβ and RegIIIγ expression in mouse intestine. Biochem Biophys Res Commun. 2020;529:64-69.

12. Busch RA, Heneghan AF, Pierre JF, Wang X, Kudsk KA. The enteric nervous system neuropeptide, bombesin, reverses innate immune impairments during parenteral nutrition. Ann Surg. 2014;260:432-444.
